# Crystal Structures, Stability, and Solubility Evaluation of a 2:1 Diosgenin–Piperazine Cocrystal

**DOI:** 10.1007/s13659-020-00256-y

**Published:** 2020-07-06

**Authors:** Ningbo Gong, Hongmei Yu, Ying Wang, Cheng Xing, Kun Hu, Guanhua Du, Yang Lu

**Affiliations:** 1 Beijing Key Laboratory of Polymorphic Drugs, Institute of Materia Medica, Chinese Academy of Medical Sciences and Peking Union Medical College, Beijing, 100050 China; 2 Beijing City Key Laboratory of Drug Target Identification and Drug Screening, Institute of Materia Medica, Chinese Academy of Medical Sciences and Peking Union Medical College, Beijing, 100050 China

**Keywords:** Diosgenin, Piperazine, Cocrystal, Characterization, Solubility

## Abstract

**Abstract:**

A cocrystal of diosgenin with piperazine in 2:1 stoichiometry was successfully synthesized. The solid form was prepared by liquid assisted grinding, slurry and crystallization methods. The cocrystal was characterized by powder X-ray diffraction, differential scanning calorimetry, thermogravimetric analysis, Fourier transform infrared spectroscopy, and structure determined by single crystal X-ray diffraction, the hydrogen bonds formed into fish bone structure along the [010] direction and all the molecules packed into 3D layer structure along a axis. After formation of cocrystal, the solubility of diosgenin was improved, and the solubility value in 0.2% SDS solution was approximately 1.5 times as large as that of the parent material.

**Graphic Abstract:**

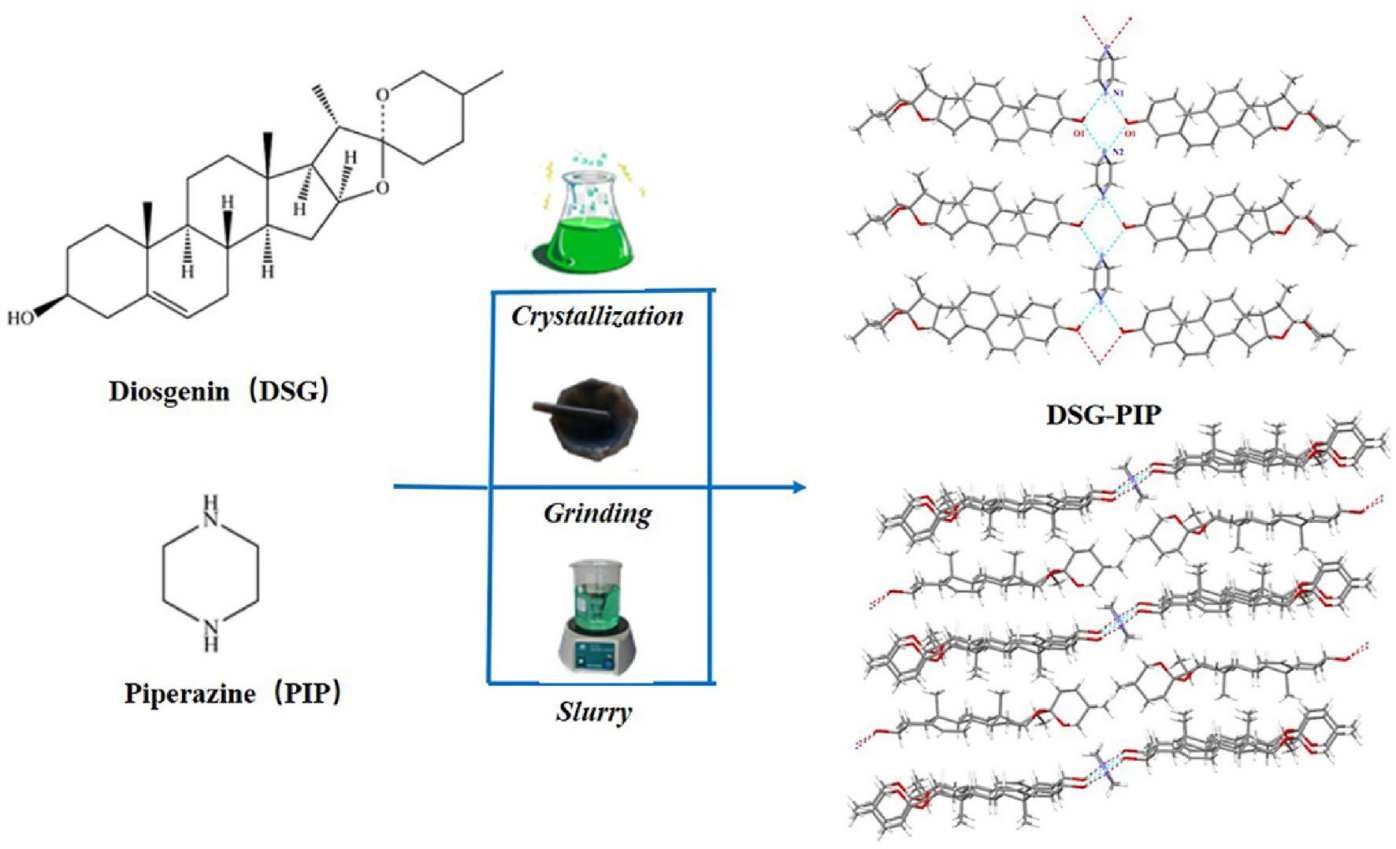

## Introduction

The solid pharmaceutical forms include polymorphs, hydrates, salts, amorphous solids, solvates, and cocrystals [1–3]. Pharmaceutical cocrystal consists of two or more molecular components containing an active pharmaceutical ingredient (API) and other solid co-crystal formers (CCFs) with a well-defined stoichiometry in the crystal lattice formed into structurally homogeneous crystalline materials [4, 5]. For new drug development, one of the main obstacles in the pharmaceutical industry is the low solubility and bioavailability. Pharmaceutical cocrystallization has been proved an effective modification mean for the target change in physicochemical properties such as the melting point, stability, hygroscopicity, solubility, and dissolution rate without altering the chemical structures and inherent bioactivities of APIs. The first FDA approved pharmaceutical cocrystal drug was Entresto comprising sacubitril and valsartan for chronic heart pain in 2015 [6–8]. Now cocrystal has attracted increasing attention in the pharmaceutical community for their desirable physicochemical and biopharmaceutical properties. The cocrystl also can be protected by legal issues with unique commercial advantages and wide development space [9]. All this encouraged pharmaceutical solid state chemists to explore more cocrystals to optimize formulation during drug development.

**Scheme 1:**
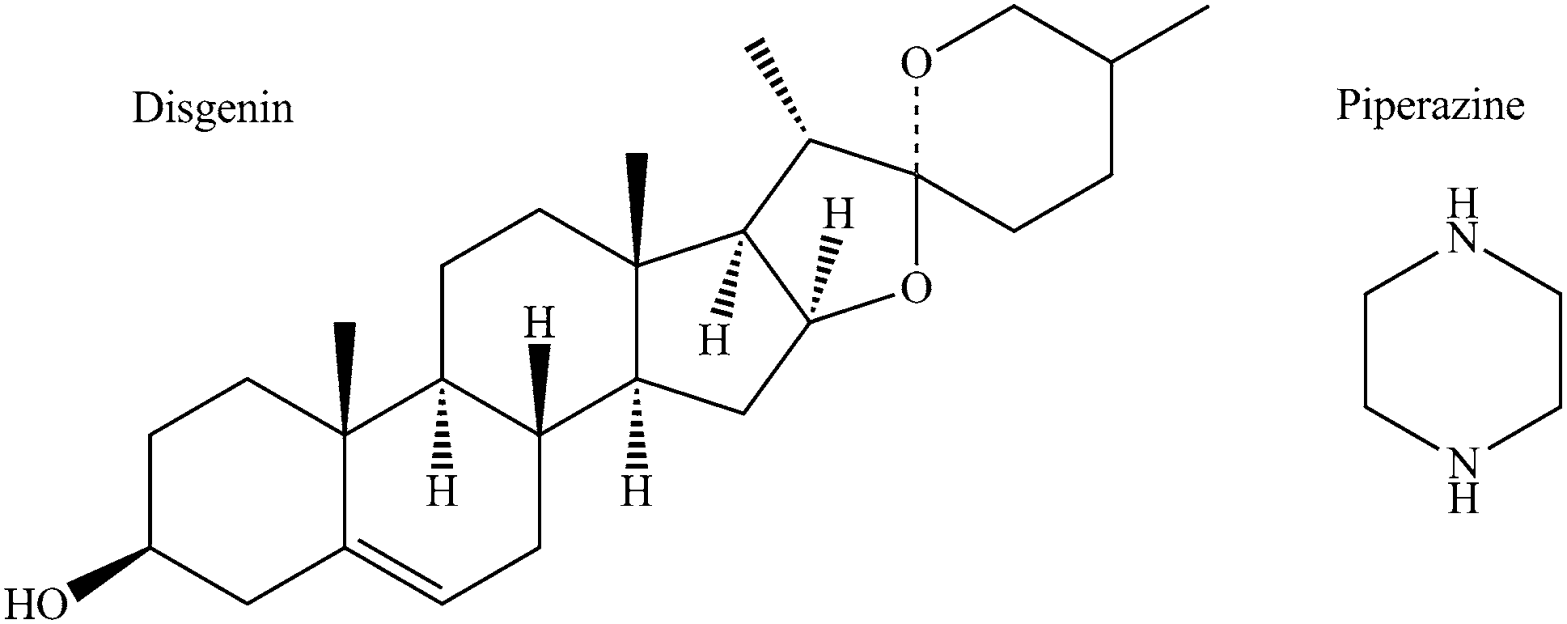
Chemical structures of diosgenin (DSG) and piperazine (PIP)

Diosgenin (Scheme 1, DSG), a well-known steroidal phytoestrogen which originated by the hydrolysis of the saponin dioscin, predominantly widely present in many plants and traditional Chinese medicines, namely, from *Dioscorea, Trigonella, Costus *[10, 11]. Diosgenin can be used as the natural precursor to commercially synthesize most of the therapeutically useful steroidal drugs, including estrogen, progesterone, testosterone, and other sex hormones or corticosteroids [12]. However, in addition to this high synthetic relevance, diosgenin itself has the benefits of antiproliferative activity [13], ameliorates testicular damage [14], anticancer activity [15, 16], anti-apoptotic effect [17], cardiovascular protective effect [18, 19], immunomodulating activity [20], among others.

Based on the literature and our previous studies [21–24], two hydrates, three hemi-hydrates and four solvates of DSG were synthesized and structurally characterized. However, the solubility was not satisfied as expected. The application of DSG as a drug in clinic was severely restricted by its poor and variable oral absorption due to its low solubility. Therefore, improving the solubility of DSG was crucial important for the drug development. In order to find new solid forms to improve the solubility, a cocrystal of DSG with piperazine (PIP) has been developed in this study. The crystal structure of the cocrystal obtained was studied in detail, and the intermolecular interactions present were analyzed. Powder X-ray diffraction (PXRD), differential scanning calorimetry (DSC), thermogravimetric analysis (TGA), Fourier transform infrared (FT-IR) spectroscopy were all used to characterize the cocrystal, and the stability and solubility were also investigated.

## Results and Discussion

### Crystal Structure Analysis

Suitable crystals were obtained by slow evaporation. The Single crystal X-ray diffraction (SXRD) results showed that the cocrystal DSG-PIP was crystallized in the monoclinic system space group C 2 and possessed 4 formula units per unit cell (Z = 4). An asymmetric unit containing DSG and PIP with the ratio 1: 0.5 emerged for this cocrystal. The crystallographic data and refinement details of the cocrystal DSG-PIP were listed in Table 1 and the CCDC number was 1997912.

**Table 1:** Crystal parameters of cocrystal of diosgenin-piperazine

	DSG-PIP cocrystal
Color/shape	Colorless/plate
Empirical formula	C_27_H_42_O_3_, 0.5(C_4_H_10_N_2_)
Molecular weight (g mol^−1^)	457.67
Crystal system	Monoclinic
Space group	C 2
Unit cell parameters ()	a = 20.373(9) Å; b = 7.482(5) Å; c = 17.541(8) Å β = 91.148(11)°
Volume (Å^3^)	2673(2)
Z	4
Density (g cm^−3^)	1.137
*F*(000)	1008
Theta range for data	4.34 < Ɵ < 72.68
Reflections collected	3789
Independent reflections	3631
Completeness (%)	98.6
Final *R*, *wR*(*F*^2^) values [*I* > 2σ(*I*)]	0.0560, 0.1469
Final *R, wR*(*F*^2^) values (all)	0.0592, 0.1568
Goodness-of-fit on F^2^	1.105
CCDC number	1997912

**Scheme 2:**
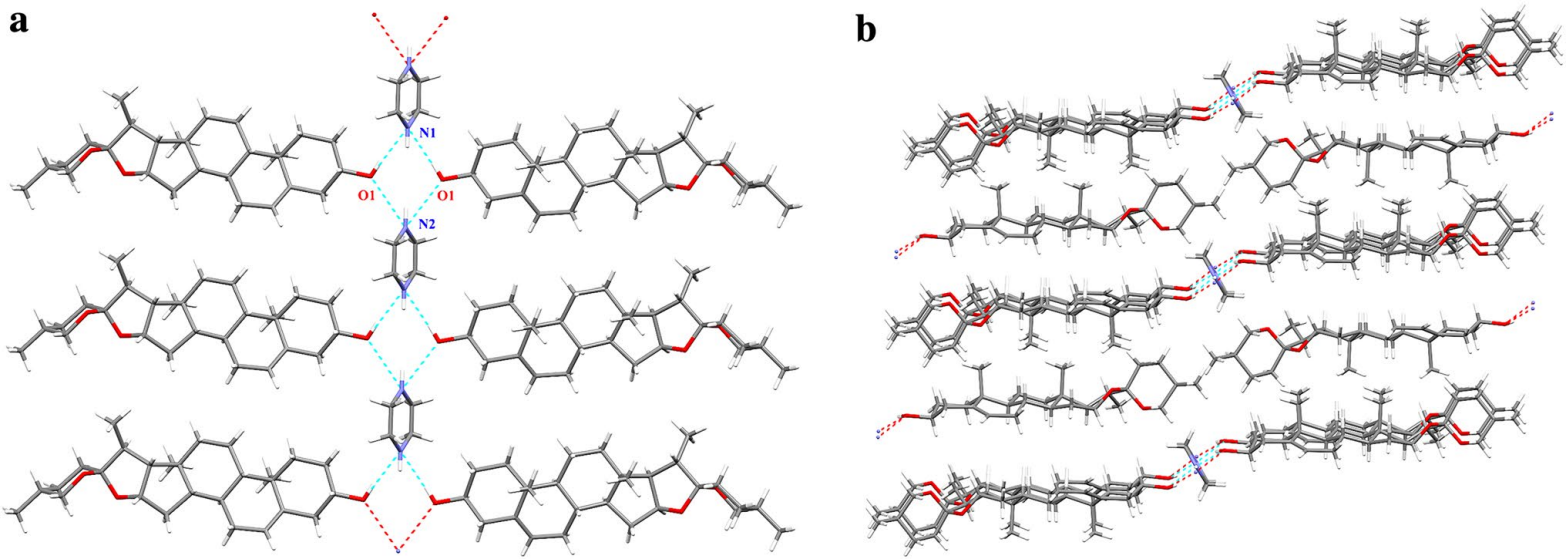
The hydrogen-bonding scheme and a view of the packing of the structure

Hydrogen bonding was one of the most utilized intermolecular interactions in crystal engineering as their directional nature lends itself to being used to control and direct the assembly process. A $R_{3}^{3}$(6) motif was found in this cocrystal by the hydroxyl group in DSG and amino group in PIP. Two hydroxyl group and two amino group contact into a ring and arrange the molecules as fish bone along the [010] direction (Scheme 2a). In addition, all the molecules packed into 3D layer structure along a axis (Scheme 2b).

### Powder X-ray Diffraction (PXRD) Analysis

**Scheme 3:**
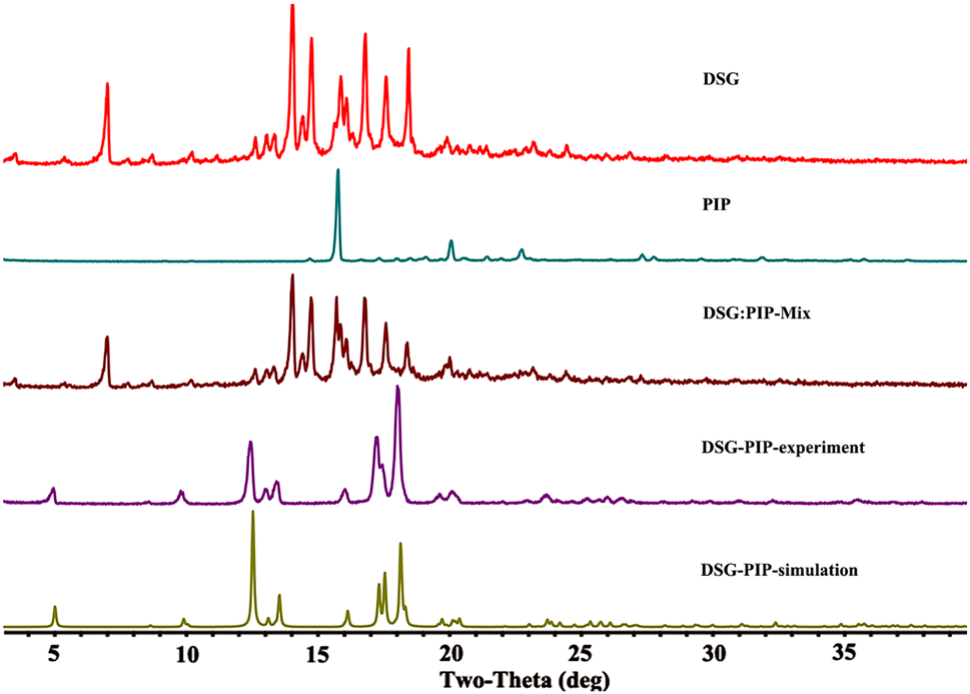
The PXRD patterns of DSG-PIP, DSG, PIP and physical mixture

Each crystalline form of a drug will produce a characteristic PXRD pattern. A simulated powder pattern calculated from SXRD data can be served as the reference pattern in the cocrystal identification. The experimental PXRD of cocrystal demonstrate good agreement with the single crystal simulated pattern (Scheme 3), which suggests that the high quality and purity of the cocrystal in a single phase. The patterns of raw materials, physical mixture (DSG:PIP-mix) were also shown in Scheme 3, which were different with the pattern of cocrystal, thereby indicating the formation of new phase.

### Thermal Analysis

**Scheme 4:**
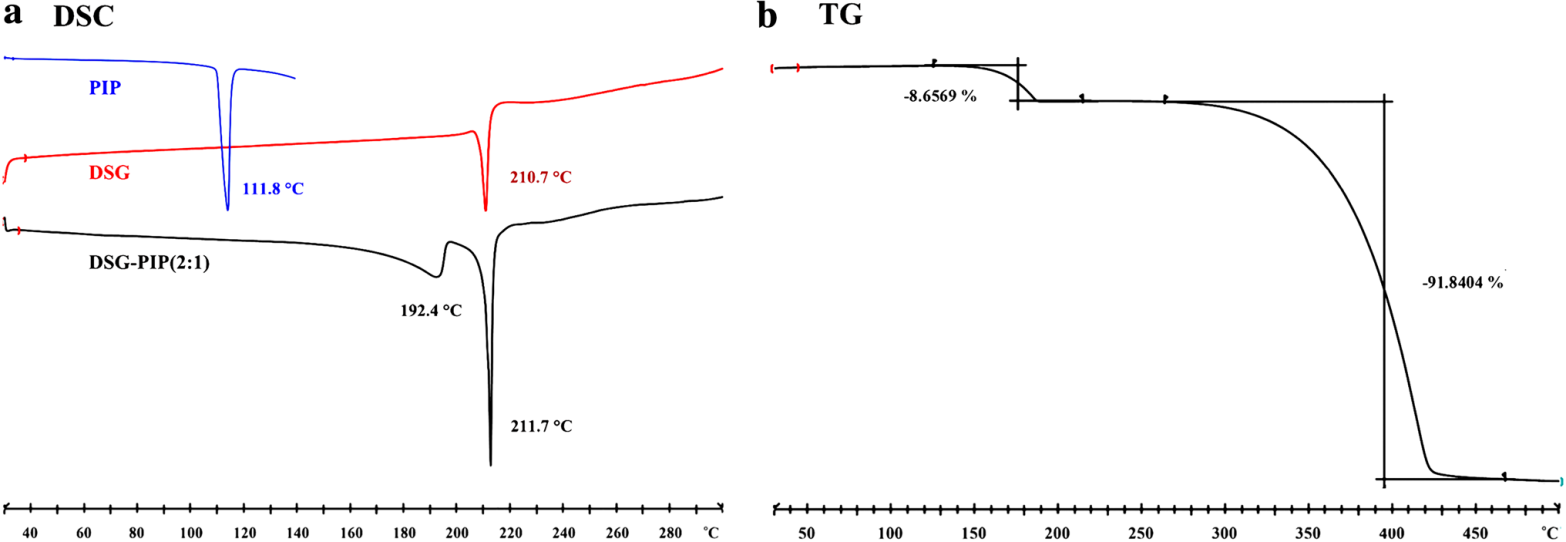
The thermal analysis patterns of DSG-PIP and raw materials

The DSC analysis was used to investigate the thermal properties of the cocrystal. From the DSC profiles (Scheme 4a), PIP showed a sharp endothermic peak at 111.8 °C, DSG showed a sharp endothermic peak at 210.7 °C. However, cocrystal showed two endothermic peak at 192.4 °C and 211.7 °C, which were different with the melt point of raw materials. This also indicating the new phase formation.

TGA was applied to investigate the decomposition process of the cocrystal. On the basis of the results of TG analysis (Scheme 4b), the cocrystal DSG-PIP did not contain any solvent or water. There were two decomposition process in the TG experiment. At first step, the mass loss was 8.7% at the temperature range 150–190 °C, the values were basically consistent with the theoretical calculation value of 9.4% ascribed as loss of 0.5 piperazine. At second step, the mass loss was 91.8% at the temperature range 300–450 °C ascribed as decomposition of diosgenin.

### Fourier Transform Infrared (FT-IR) Spectroscopy Analysis

**Scheme 5:**
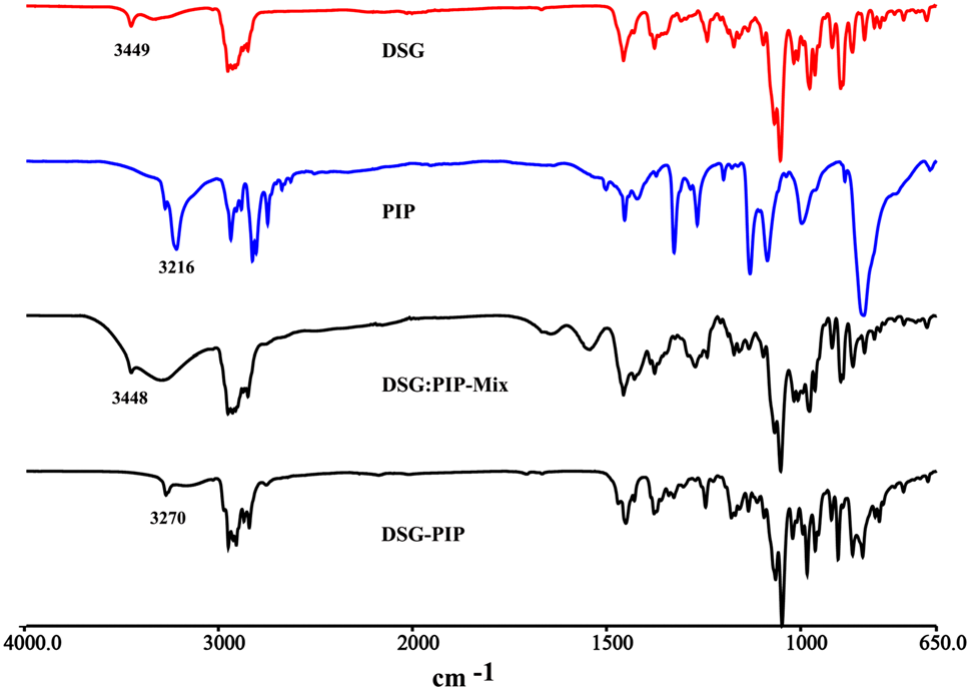
The FT-IR patterns of DSG-PIP, DSG, PIP and physical mixture

In the FT-IR pattern (Scheme 5), the vibrational absorption peaks of hydroxyl group and the characteristic absorption bands of spiroketal skeleton were almost the same in the cocrystal and DSG. However, the 3000–3500 cm^−1^ band owing to the hydrogen-bonding patterns of hydroxyl groups and amino group in the cocrystal were obviously different form the corresponding starting material. So infrared spectroscopy can be used as a valuable supplementary tool to identify the new solid phase of DSG-PIP.

### Stability Studies

**Scheme 6:**
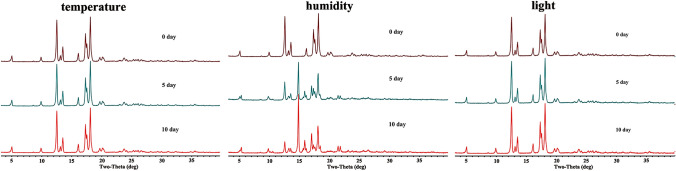
Stability of DSG-PIP under three conditions

PXRD was used to evaluate the stability of DSG-PIP under three conditions, temperature (40 ± 1 °C); humidity (90% ± 5% RH, 25 °C), light (4500 lx ± 500 lx, 25 °C)( Scheme 6). DSG-PIP was stable under temperature and light condition for 10 days, respectively. Nevertheless, the cocrytsal was unstable under humidity condition, which indicated that the humidity should be take care during the storage and formulation process.

### Solubility and Dissolution Studies

**Scheme 7:**
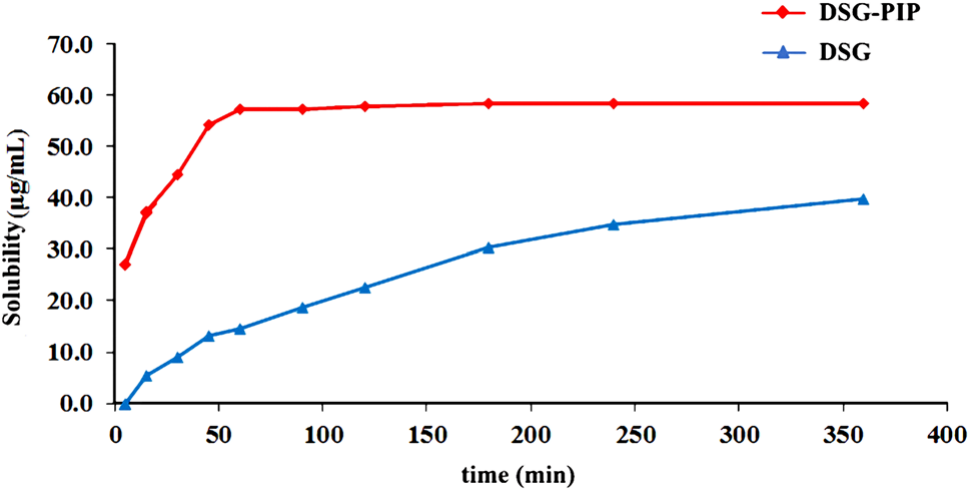
Concentration–time curves of DSG-PIP and DSG

Due to the poor solubility of diosgenin in water, 0.2% SDS solution was used as the incubation media to test the solubility and dissolution process. The time-solubility profiles of diosgenin and cocrystal in 0.2% SDS solution were presented in Scheme 7. The solubility of DSG-PIP was 58.5 μg mL^−1^, and was approximately 1.5 times as high as that of parent DSG (39.9 μg mL^−1^). The solubility of DSG-PIP was obviously higher than that of DSG, which can be attributed to the cocrystal formation. Similarity factor (ƒ_2_) method was used to evaluate the similarity of dissolution curve of DAG-PIP and DSG. Only the calculated ƒ_2_ is between 50 and 100, the products are considered similar. The ƒ_2_ value in the dissolution study was 15.30 indicates obvious difference between two dissolution curves of cocrystal and raw material. DSG-PIP achieves a higher DSG concentration with a faster rate. The dissolution plot reached the maximum apparent solubility (*S*max) within 50 min, which was faster than that of DSG. This given us a hint that DSG could directly form a cocrystal with suitable CCF with good solubility to improve the solubility and dissolution rate.

In conclusion, a diosgenin–piperazine cocrystal was successfully prepared by three different methods (liquid assisted grinding, slurry and crystallization) for the first time. The single crystal structure analysis showed diosgenin and piperazine were combined together with 2:1 chemical stoichiometry. Piperazine molecules acted as intermolecular hydrogen-bonding acceptors and donors, were manifested to explicitly or implicitly affect the intermolecular arrangement and therefore stabilize crystal lattices by fostering stronger interactions and lower the energy of the system. The PXRD, DSC, TGA, FT-IR methods could all be used to identify and characterize the cocrystal. DSG-PIP showed stable at temperature (40 ± 1 °C) and light (4500 lx ± 500 lx, 25 °C) conditions; but unstable at humidity (90% ± 5% RH, 25 °C) for 10 days. In addition, cocrystal exhibit higher solubility and faster dissolution speed than pure DSG. Taking into account the above fact, it is of considerable interest to synthesize and study cocrysal of DSG and piperazine. Pharmaceutical cocrystallization technique not only can provide an opportunity to find new solid forms of drugs, but also can offer a route to optimize physicochemical and pharmacokinetic properties of APIs, which makes it practical to fast develop new drugs. With further study, these findings may provide opportunities to exploit a novel pharmaceutical combination in the future.

## Experimental Section

### Materials

Diosgenin raw material was purchased from Wuhan Yuancheng Science and Technology Development Co., Ltd. (Hubei Province, China). Methanol (HPLC grade) and acetonitrile (HPLC grade) were supplied by Thermo Fisher Scientific Company (Shanghai, China). All analytical grade solvents were purchased from the Sigma Aldrich Reagent Company and were used without further purification.

### Cocrystal Preparation and Crystallization

DSG-PIP cocrystal was obtained using different preparation methods as described below.

#### Liquid Assisted Grinding Method

40 mg of DSG and 4.16 mg of PIP were ground using a mortar and pestle for 10 min with addition of a few drops of acetone. The resulting powders were collected for characterization.

#### Slurry Method

400 mg of DSG and 41.55 mg of PIP were suspended in ethanol. The suspension was agitated at 400 g for 24 h at room temperature, and the agglomerates were filtered and dried at 40 °C under vacuum for 12 h.

#### Crystallization Method

40 mg of DSG and 4.16 mg of PIP were dissolved in the minimum amount of ethanol. The solutions were allowed to evaporate slowly at room temperature for about 10–15 days until crystals suitable for single crystal X-ray diffraction experiments were obtained.

### Single Crystal X-ray Diffraction Analysis

Single crystal X-ray diffraction data of DSG-PIP was collected on a MicroMax 002+ diffractometer equipped with Cu-Kα radiation (λ = 1.54187 Å). Cell refinement and data reduction were applied using the program of CrystalClear (Rigaku Inc., 2008). The crystal structures were solved by direct methods using SHELXS-2016 [25] and refined by full-matrix least-squares refinement on F^2^. Hydroxyl H atoms were located from the difference electron density maps. Other H atoms were refined isotropically and were placed in calculated positions using riding models.

### Powder X-ray Diffraction Analysis

PXRD patterns were obtained using a D/max-2550 (Rigaku, Japan) X-ray diffractometer (λ = 1.54187 Å) with voltage and current set 40 kV and 150 mA, respectively. Samples were measured in reflection mode in the 2θ range 3–40° with a scan speed of 8° min^−1^ (step size 0.02°) at room temperature. Data were analyzed using MDI Jade 6.5 software. The simulated powder pattern was calculated from the single crystal data using Mercury 4.3.1.

### Thermal Analysis

DSC and TGA measurements of the cocrystal were performed on a Mettler Toledo DSC 1 module and a Mettler Toledo TGA/DSC STARe module, respectively. Sample weighing about 3–10 mg was placed in aluminum sample pans and was heated at 10 ºC min^−1^ over a temperature range from 30 to 300 °C under dry nitrogen atmosphere (with a flow rate of 50 cm^3^ min^−1^) in the DSC test. The DSC apparatus was calibrated using Indium as the reference material and the TGA/DSC data were analyzed by using STARe software.

### Fourier Transform Infrared Spectroscopy Analysis

FT-IR spectra were collected in a PerkinElmer Spectrum 400 FT-IR spectrophotometer (PerkinElmer, U.S.) and recorded in the spectral range of 4000–650 cm^−1^ with a 4 cm^−1^ resolution. An attenuated total reflectance (ATR) sampling accessory with a diamond window was used for measurements.

### Stability Studies

All solid-state forms of DSG-PIP were stored in a drug stability test instrument (SHH-150SD) at three conditions, temperature (40 ± 1 °C); humity (90% ± 5% RH), light (4500 lx ± 500 lx), respectively. Periodically, samples were removed from the instrument and subjected to PXRD testing to explore the changes of the systems.

### Solubility and Dissolution Profile Studies

Solubility experiments were carried out by the shake-flask method. Suspensions of the respective cocrystal and diosgenin in 100 mL of a 0.2% SDS solvent in erlenmeyer flasks were shaken in a table concentrator (Shaking Incubator ZHWY-103D Labwit Scientific Pty Ltd, China) for hours at 25 ± 0.1 °C. The dissolved amount of diosgenin was determined by high performance liquid chromatography (HPLC) using a HPLC instrument (Agilent 1200, Agilent, USA) at definite intervals (5 min, 15 min, 30 min, 45 min, 60 min, 90 min, 120 min, 180 min, 240 min, 360 min) at the reference wavelength (210 nm). The absorbance values were correlated to solution concentrations using a calibration curve. The samples were separated by using an Aligent XDB-C18 5 μm (150 mm × 4.6 mm) column. The mobile phase consisted of acetonitrile and methanol (40:60, v/v) eluted at 1.0 mL min^−1^. The column temperature was 30 °C and the injection volume was 10 μL.
